# Prognostic Significance of the Systemic Inflammatory and Immune Balance in Alcoholic Liver Disease with a Focus on Gender-Related Differences

**DOI:** 10.1371/journal.pone.0128347

**Published:** 2015-06-24

**Authors:** Beata Kasztelan-Szczerbińska, Agata Surdacka, Krzysztof Celiński, Jacek Roliński, Agnieszka Zwolak, Sławomir Miącz, Mariusz Szczerbiński

**Affiliations:** 1 Department of Gastroenterology with Endoscopy Unit, Medical University of Lublin, Lublin, Poland; 2 Department of Clinical Immunology, Medical University of Lublin, Lublin, Poland; 3 Medical University of Lublin, Lublin, Poland; 4 Department of Gastroenterology, Provincial Specialist Hospital, Lublin, Poland; University of Sydney, AUSTRALIA

## Abstract

**Objectives:**

Mechanisms of immune regulation in alcoholic liver disease (ALD) are still unclear. The aim of our study was to determine an impact of Th17 / regulatory T (Treg) cells balance and its corresponding cytokine profile on the ALD outcome. Possible gender-related differences in the alcohol-induced inflammatory response were also assessed.

**Materials and Methods:**

147 patients with ALD were prospectively recruited, assigned to subgroups based on their gender, severity of liver dysfunction and presence of ALD complications at admission, and followed for 90 days. Peripheral blood frequencies of Th17 and Treg cells together with IL-1beta, IL-6, IL-17A, IL-23, and TGF-beta1 levels were investigated. Flow cytometry was used to identify T cell phenotype and immunoenzymatic ELISAs for the corresponding cytokine concentrations assessment. Multivariable logistic regression was applied in order to select independent predictors of advanced liver dysfunction and the disease complications.

**Results:**

IL-17A, IL-1beta, IL-6 levels were significantly increased, while TGF-beta1 decreased in ALD patients. The imbalance with significantly higher Th17 and lower Treg frequencies was observed in non-survivors. IL-6 and TGF-beta1 levels differed in relation to patient gender in ALD group. Concentrations of IL-6 were associated with the severity of liver dysfunction, development of ALD complications, and turned out to be the only independent immune predictor of 90-day survival in the study cohort.

**Conclusions:**

We conclude that IL-6 revealed the highest diagnostic and prognostic potential among studied biomarkers and was related to the fatal ALD course. Gender-related differences in immune regulation might influence the susceptibility to alcohol-associated liver injury.

## Introduction

Alcoholic liver disease remains a major health problem worldwide and its prevalence is increasing in Eastern, as well as some Western countries (eg. United Kingdom, Ireland, Finland) [1]. Furthermore, alcohol consumption accounts for 3.8% of global mortality [2]. Consequently, ALD should remain in focus of extensive research in hepatology.

Significant progress has been made in our understanding of the role of inflammatory activation and immune dysregulation in deterioration of the disease course and its outcome. Nevertheless, the underlying mechanisms are still poorly understood. It is widely accepted that both acute and chronic ethanol consumption alters the immune system function [3, 4, 5, 6]. Ethanol may impair antigen presentation by monocytes and dendritic cells, interfere with the expression of adhesion molecules and decrease T cell proliferation [7]. In parallel with ALD progression and increasing synthesis of pro-inflammatory cytokines/ chemokines by Kupffer cells, an immune response towards antigens derived from damaged hepatocytes and massive inflammatory cell recruitment into the liver may occur [8].

Accumulating evidence highlights the importance of two new subsets of CD4+T cells, Th17 and regulatory T (Treg) cells in the pathogenesis of several autoimmune and inflammatory disorders. We speculate that ALD might be one of them, but to date, the Th17/Treg balance has not been explored in patients with ALD yet. It is believed that Th17 and Treg cells are critically linked to inflammatory immune response [9–12]. While Th17 lymphocytes exert pro-inflammatory effects, Treg cells are potent immune suppressors, and they may reciprocally control their function. Human Th17 differentiation is IL-1beta1, IL-6, IL-23 and IL-17A- dependent and suppressed by TGF-beta1. On the other hand TGF-beta1 enhances the differentiation of human Treg cells [13–17].

Moreover, clinical observations indicate that women develop more severe alcohol-associated liver injury at lesser ethanol intake and fewer years of exposure. So far underlying mechanisms remain obscure. Females have a stronger inflammatory response in comparison to males [18, 19]. These differences are believed to have an association with different hormone patterns (estrogens are immune stimulators, testosterone is rather immunosuppressive). Therefore, the inflammatory response in the course of ALD may differ in men and women.

Accordingly, we aimed to assess the significance of peripheral Th17/Treg balance and corresponding cytokine profile in the course of ALD. We hypothesized that their possible alterations (a consequence of the local and systemic inflammatory response) might affect the disease progression and development of its complications. In addition, we investigated whether gender-related differences in the alcohol-induced inflammatory and immune response existed and how they might influence the disease course.

Our prospective study was conducted in the Department of Gastroenterology with Endoscopy Unit of Medical University in Lublin, Poland.

## Materials and Methods

### Patient characteristics

The study cohort has been described in detail in our previously published report concerning adipose tissue biomarkers [20].

Briefly, 147 unselected and consecutive adult inpatients (pts) (40 females, 107 males) with ALD were prospectively recruited over a 2-year period and followed for 90 days.

The control group consisted of 30 age- and sex-matched healthy volunteers (13 females, 17 males), who pledged abstinence or alcohol consumption as no more than 20 g ethanol per day. The diagnosis of ALD was established by combination of typical symptoms and physical findings of chronic liver disease, as well as laboratory results i.e. elevated aminotransferase activity (normal range: ALT < 31IU/L; AST< 34IU/L), the AST/ALT ratio above 2, and imaging studies in the setting of excessive alcohol intake. Other cofactors of chronic liver injury were excluded.

Alcohol abuse was confirmed by the AUDIT-C (Alcohol Use Disorders Identification Test—Consumption) questionnaire [21]. The positive result of AUDIT-C, in addition to the amount of alcohol intake, was an inclusion criterion. The daily alcohol consumption in the study group ranged as follows: in females 40 g/d to more than 100 g/d, in males 50 g/d to more than 100 g/d.

Neither corticosteroids nor pentoxifylline were administered to any individuals at the time of enrollment.

Patients demographic data as well as all procedures were recorded and performed within 48 hours after hospital admission. Blood samples were collected at 07:30 am after a minimum 8- hour overnight fast.

The severity of liver failure at baseline was established using the Child-Turcotte-Pugh (CTP) [22] and MELD (the Model of End-Stage Liver Disease) [23] criteria. Patients were assigned into different subgroups according to their:

1. gender,
2. the severity of liver dysfunction according to the CTP (class A, B, C) and MELD (≥ 20 or < 20) scores;
3. the presence of ALD complications at the time of hospital admission i.e. ascites, hepatic encephalopathy (HE), oesophageal varices, cholestasis, and renal impairment.

Subjects with severe comorbidities present at the time of enrollment i.e. malignancy, pulmonary insufficiency, heart failure or uncontrolled diabetes were excluded.

Symptoms of overt hepatic encephalopathy (HE) were classified according to West-Heaven criteria [24].

Abdominal ultrasonography was performed to confirm the presence of ascites and gastroscopy in order to identify esophageal varices.

The normal values for creatinine in the blood range from 0.6 to 1.3 milligrams per deciliter in our laboratory, therefore its level above 1.3 mg / dL (the upper limit of normal) was considered a criterion of renal impairment.

All enrolled patients were discharged from hospital once alcohol withdrawal symptoms have disappeared and liver function has begun to improve. Subsequent follow-up visits during next 90 days were set at least once a month (generally every 2 weeks) in the outpatient clinic or during any hospital admission if required. Two of nonsurvivors returned to our department after their condition worsened and they died in hospital. The majority of nonsurvivors (10 of 12) were treated continuously without any hospital discharge.

### Laboratory examinations

Analysis of basic laboratory tests included: liver function parameters, complete blood count, parameters of renal function and traditional markers of inflammation (neutrophils, neutrophil to lymphocytes count rate (NLR) and the level of C-reactive protein- CRP).

In addition, the evaluation of uncommon inflammatory indicators and immune regulators in peripheral blood was performed. We determined frequencies and the systemic balance of two new subsets of T helper cells: Th17 and regulatory T lymphocytes (Treg), as well as concentrations of corresponding interleukins (IL) which were reported to be the hallmarks of human Th17 and Treg lymphocyte differentiation, this is IL-1beta, IL-6, IL-17A, IL-23 and Transforming Growth Factor beta 1 (TGF-beta1), respectively.

### Cell isolation, culture and flow cytometric analysis

Peripheral blood mononuclear cells (PBMC) were isolated from 10 ml of heparinized venous blood samples. For Th17 frequency detection, four hour cultures of PBMC in the presence of stimulators i.e. ionomycin (1μg/ml) (Salt Cacium Ionomycin from Streptomyces, Sigma-Aldrich) and phorbol myristate acetate-PMA (50 ng / ml) (Sigma-Aldrich, USA), as well as 10 μg/ml of brefeldin A (Sigma-Aldrich, UK), were employed. Cells were labeled with fluorescein isothiocyanate (FITC)-conjugated anti-human CD 3 and CD4 antibodies directed against cell surface antigens (anti-human CD3 PE-Cy5, anti-human CD4 FITC, BD Biosciences). Then, the samples were fixed, permeabilized and stained with the intracellular phycoerythrin (PE)-conjugated anti-human IL-17A (anti-IL-17-PE, human, Miltenyi Biotec) according to the protocol provided by the manufacturer (eBiosciences, San Diego, USA).

For determination of the Treg cell frequency (CD4^+^CD25^high^FoxP3^+^), Human Treg Flow TM Kit (FoxP3 Alexa Fluor 488/CD4 PE-Cy5/CD25 PE, BioLegend) was used in accordance with the recommendations of the manufacturer. Firstly, peripheral blood samples (100 μL) were surface-stained with FITC-conjugated anti-human CD4 antibodies and allophycocyanin (APC)-conjugated anti-human CD25 antibodies for 30 minutes, then treated with Fix/Perm buffer (eBiosciences, USA) according to the manufacturer’s instructions. Intracellular FoxP3 expression was determined using mouse anti- FoxP3 Alexa Fluor 488 at concentration of 5 ml per tube, for 30 minutes, in the dark at room temperature. After the intracellular staining, cells were washed in 0.5 ml of buffer and evaluated for the expression of FoxP3 and CD25 on CD4^+^ cells by flow cytometry. Each sample was carried out in parallel to the negative control using isotypes for anti-FoxP3.

**Fig 1:**
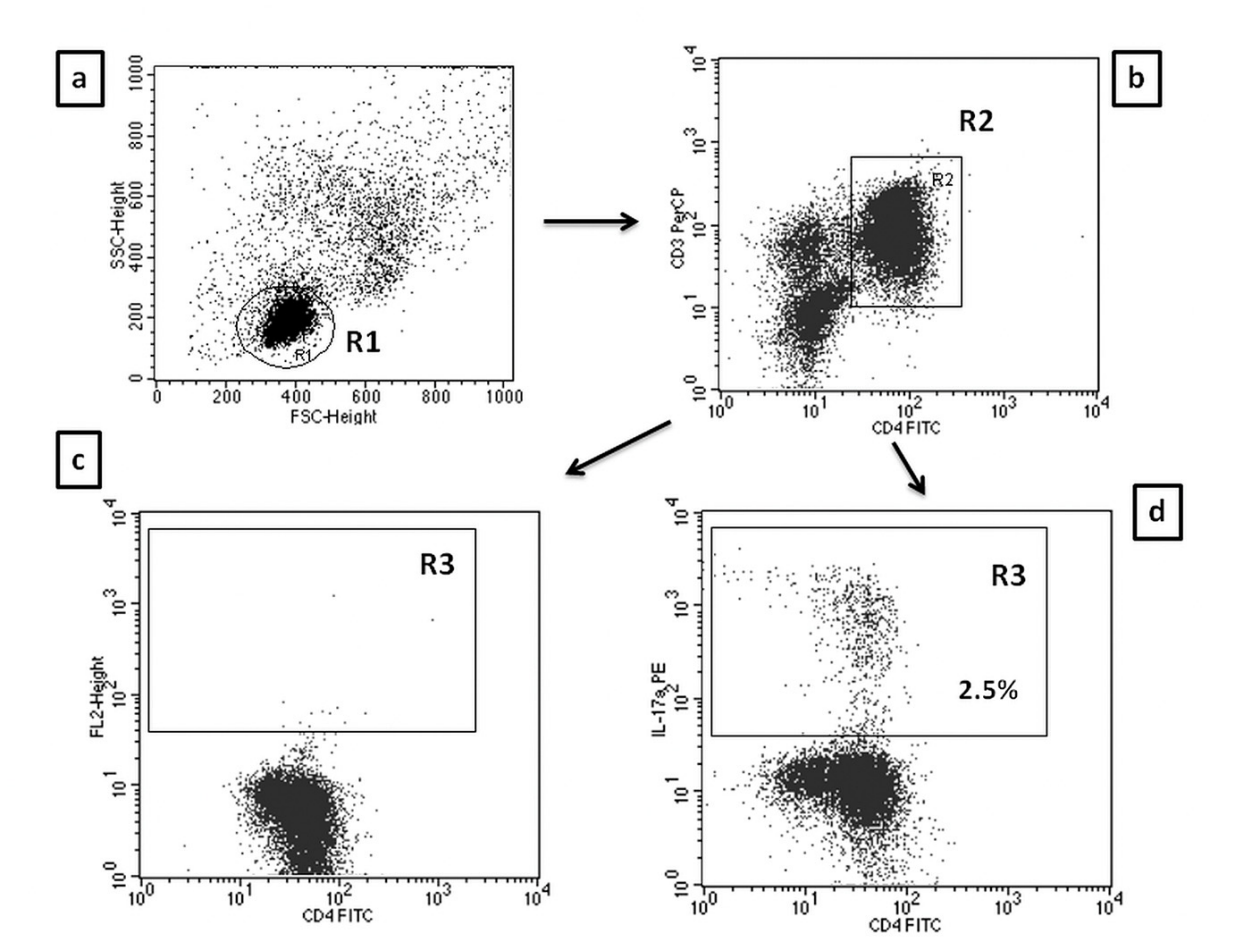
Evaluation of CD4/CD3+/IL-17A+ (Th17) cells frequency in ALD patients by flow cytometry. A. The lymphocyte subpopulation was gated (R1) based on linear forward vs. side scatter (FSC/SSC) characteristics. B. The R1 cells were further evaluated for CD3 PE-Cy5 and CD4 FITC staining with subsequent CD4+/CD3+ cell identification (region R2). C. Dot plots of CD4FITC versus mouse IgG1 PE were established (controls). D. Dot plots of CD4FITC versus IL-17A PE were established by combined gating of R1 and R2 events. The R3 region represents the percentage of CD4+/CD3+/IL-17A+ (Th17) cells among all CD4+CD3+ lymphocytes.

**Fig 2:**
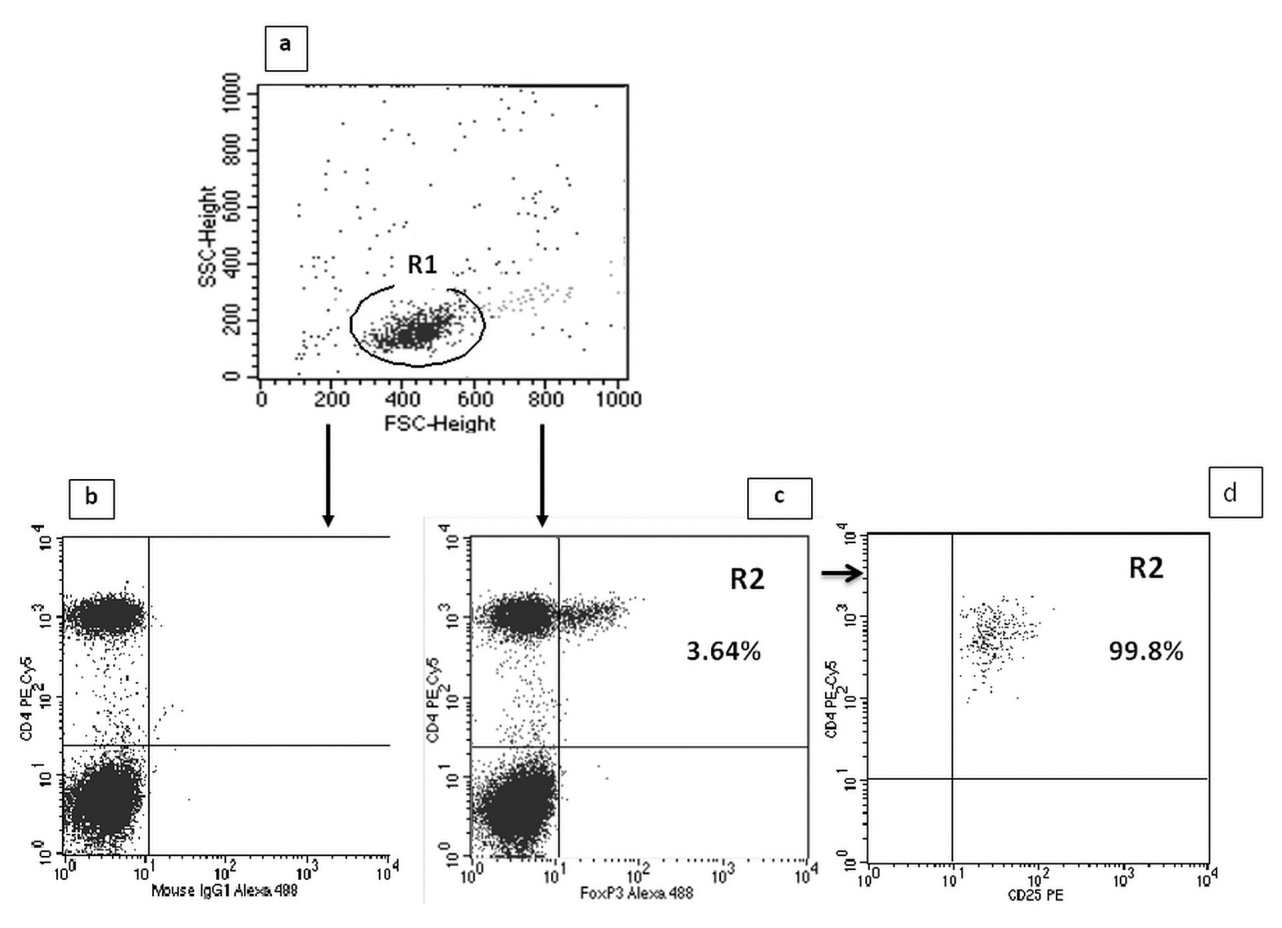
Evaluation of CD4^+^CD25^high^FoxP3^+^ (Treg) cells frequency in ALD patients by flow cytometry. A. The lymphocyte subpopulation was gated (R1) based on linear forward vs. side scatter (FSC/SSC) characteristics. B. Dot plots of CD4 PE-Cy5 versus mouse IgG1 Alexa Fluor 488 were established (controls). C. Dot plots of CD4 PE-Cy5 versus the intracellular expression of FoxP3 Alexa Fluor 488 in CD4+ T cells were established. The R2 region represents the percentage of CD4+CD25^high^FoxP3+ (Treg) cells among all CD4+CD25+ lymphocytes. D. 99.8% of cells gated as CD4+FoxP3+ (R2) were CD25 ^high^.

A FACSCalibur flow cytometer (Becton Dickinson, USA) with CellQuest Pro software was used to identify T cell phenotype and to perform analyses. Isotype-matched antibodies were used for verification of staining specificity and for setting the markers to delineate positive and negative populations. Percentage of cells positively labeled with monoclonal antibodies was estimated. Th17 cells were characterized as CD3^+^CD4^+^IL17^+^ and Tregs as CD4^+^CD25^high^FoxP3^+^, and expressed as the percentage of all CD3^+^CD4^+^ and CD4^+^CD25^+^ lymphocytes, respectively. Examples of cytometric evaluation of Th17 and Treg cells are presented in Figs 1 and 2.

### Determination of serum interleukin concentrations

The concentrations of selected cytokines: IL-1beta, IL-6, IL-17A,, IL-23, TGF-beta were measured using commercially available ELISA kits (Quantikine ELISA kit, R&D Systems, USA). Blood samples were obtained by venipuncture into vacutainer tubes containing EDTA (Medlab, Great Britain) and centrifuged within 30 minutes for 15 minutes at 4°C. Plasma samples were stored frozen at ≤ −80°C until the time of estimation. The examination was conducted according to the procedure recommended by the producer and described in the attached materials. Measurements were performed using VictorTM3 Reader (PerkinElmer, USA).

### Statistical analysis

Statistical analysis was performed using the Statistica 10 software package (StatSoft, Poland). The distribution of the data in the groups was preliminarily evaluated by Kolmogorow and Smirnov test. A skewed distribution of checked values was found, so continuous variables were presented as medians with interquartile range and assessed using Mann-Whitney U test. Categorical variables were described as numbers with percentage and compared using either Fisher’s exact test or the χ^2^ test as appropriate. The differences in inflammatory and immune biomarker levels between CTP classes were analyzed using Kruskal-Wallis and multiple comparisons post-hoc tests. Spearman’s rank correlation test was used for the assessment of association between parameters of liver function, traditional indicators of inflammation and new immune biomarker plasma levels. The receiver operating curves (ROC) for significant factors were constructed and their areas under the curve (AUCs) checked in order to assess their accuracy in predicting the degree of liver failure and the development of ALD complications. The method of DeLong et al. [25] for the calculation of the Standard Error of the AUC was used. The Youden index and its associated cutoff point was estimated for each marker [26]. Then, multivariable logistic regression was applied in order to select independent predictors of advanced liver dysfunction and the development of ALD complications with adjustment for the patient background. A two- sided p- value of less than 0.05 were considered to be associated with statistical significance.

### Ethical requirements

All individuals signed the written informed consent prior to their inclusion in the study and were free to withdraw at any time without providing a reason. Strict confidentiality was maintained throughout the process of data collection and analysis. The study protocol conforms to the ethical guidelines of the 1975 Declaration of Helsinki (6th revision, 2008) as reflected in a priori approval by the institutional review board of Medical University of Lublin (KE-0254/141/2010).

## Results

### Significant differences in the basic characteristics between males and females with ALD

One hundred and forty seven patients (pts) met the inclusion criteria, 107 males (72.8%) and 40 females (27.2%). Their mean age was 49.84 ± 11.53 and 48.82 ± 9.94, respectively. Twelve (8.16%) of 147 pts with ALD died from complications of liver failure within 90 days of follow up. The matching control group consisted of 17 (56.7%) males and 13 (43.3%) females aged 44.31 ± 10.23 and 43.11 ± 8.43, respectively. Several surveys have indicated that the relative risk of alcohol- associated liver injury is higher in females in comparison to males. [27–29]. Therefore, patients were assigned to two subgroups based on their gender. The baseline characteristics of ALD patients is summarized in Table 1.

**Table 1:** Demographic and laboratory data in patients with ALD.^a^

	ALD group (n = 147)				
	Females (n = 40)		Males (n = 107)		
	median	95% CI	median	95% CI	p
Age years	51.00	48.03–54.96	51.00	48.00–52.49	0.19
ALT IU/L	39.50	28.03–44.93	56.00	50.00–69.00	**0.004**
ASP IU/L	100.50	78.45–114.90	110.00	78.51–131.00	0.72
AP IU/L	118.50	111.68–156.27	129.00	118.00–148.00	0.62
GGT IU/L	415.00	174.00–543.00	359.00	200.50–504.88	**0.020**
Bil mg/dL	4.20	3.51–5.27	3.00	1.75–4.00	0.70
Alb g/dL	3.10	2.70–3.29	3.20	3.00–3.30	0.12
INR	1.45	1.39–1.64	1.21	1.16–1.30	**0.034**
Crea mg/dL	0.80	0.70–0.80	0.90	0.90–1.00	0.51
Na mEq/L	139.00	136.03–140.96	138.00	136.51–139.00	0.38
Hgb g/dL	11.20	10.34–11.50	12.10	11.60–12.70	**<0.001**
RBC x10^6^kom/uL	3.17	3.08–3.50	3.86	3.57–3.97	**<0.001**
PLT x10^3^kom/uL	135.50	114.38–137.96	136.00	116.00–166.46	0.81
WBC x10^3^kom/uL	8.12	5.42–11.63	7.12	6.30–8.28	0.75
NEUT x10^3^kom/uL	8.44	3.20–8.97	5.02	4.19–6.10	**0.053**
NLR	4.38	2.34–4.52	3.47	3.26–4.45	0.12
CRP mg/L	17.33	16.19–33.14	17.53	13.40–21.30	0.58
mDF	17.35	12.00–22.96	9.00	6.00–12.00	0.21
MELD	17.50	15.03–18.00	15.00	14.00–16.00	**0.047**
CTP	9.50	9.00–10.00	7.00	7.00–8.00	**<0.001**
IL-17A pg/mL	9.06	8.23–11.12	12.11	10.19–14.47	0.41
IL-1beta pg/mL	1.50	0.56–2.94	0.65	0.37–1.34	0.13
IL-6 pg/mL	27.32	26.45–36.15	19.88	14.77–24.91	**0.02**
IL-23 pg/mL	9.63	6.08–21.75	11.80	7.77–17.38	0.58
TGF-beta1 pg/mL	507.160	305.70–701.53	617.17	388.10–711.21	**0.05**
Th17%	0.92	0.65–1.05	0.91	0.72–1.12	0.54
Treg %	3.49	2.99–4.70	3.03	2.70–3.59	**0.07**

^a^Alb- albumin (normal range (NR) 3.2–4.8); ALD- alcoholic liver disease; ALT- alanine aminotransferase (NR) < 31); AP- alkaline phosphatase (NR 45–129); AST- aspartate aminotransferase (NR < 34); Bil- bilirubin (NR 0.3–1.2); CI-Confidence Interval; Crea- creatinine (NR 0.5–1.1); CRP- C-reactive protein (NR 0.0–5.0); CTP- Child-Turcotte- Pugh score; GGT- gamma-glutamyl transpeptidase (NR <50.0); Hgb- hemoglobin (NR 14.0–18.0); IL- interleukin; INR- International Normalized Ratio (NR 0.8–1.2); MELD- Model for End-Stage Liver Disease; Na- sodium (NR 136–145); NEUT- neutrophils (NR 1.8–7.7); NLR- neutrophil to lymphocyte ratio; PLT- platelets (NR 130–400); RBC- red blood cells (NR 4.5–6.1); TGF- Transforming Growth Factor; Th- T helper cells; Treg- regulatory T cells; WBC- white blood cells (NR 4.8–10.8).

Men and women in our cohort differed significantly with respect to liver function parameters. Females presented with significantly lower activity of ALT, but higher GGT and INR and consistently more severe liver dysfunction as indicated by both MELD and CTP scores. Also anemia was more prevalent in women with ALD in comparison with men. Moreover, significant differences of IL-6 and TGF-beta1 levels and the tendency to the higher percentage of Treg cells in females were observed.

### Pro- inflammatory cytokine profile and its gender-related differences in patients with ALD

Concentrations of four tested cytokines in ALD group significantly differed in comparison to controls. Plasma IL-17A, IL-1beta, IL-6 levels were increased, while the level of TGF-beta1 was significantly lower. Results are presented in Table 2.

**Table 2:** Comparison of immune regulator levels in ALD patients and the control group.^a^

	Immune regulators				
	ALD group n = 147		Control group n = 30		
	median	95% CI	median	95% CI	p
**IL-17Apg/mL**	10.52	9.65–12.32	6.53	4.08–10.02	**<0.001**
**IL-1beta pg/mL**	0.73	0.50–1.30	0.34	0.05–1.31	**0.050**
**IL-6 pg/mL**	21.50	17.90–26.87	0.35	0.35–0.42	**<0.0001**
**IL-23 pg/mL**	10.80	8.32–15.29	16.38	6.87–24.86	0.71
**TGF-beta1 pg/mL**	553.59	458.13–635.24	840.45	660.27–1951.17	**0.006**
**Th17%**	0.92	0.78–1.05	0.98	0.74–1.32	0.66
**Treg %**	3.67	2.92–3.59	3.46	2.51–4.17	0.96

^a^ ALD- alcoholic liver disease, CI- Confidence Interval, IL- interleukin, p- level of significance, TGF- Transforming Growth Factor, Th- T helper cells; Treg- regulatory T cells.

**Fig 3:**
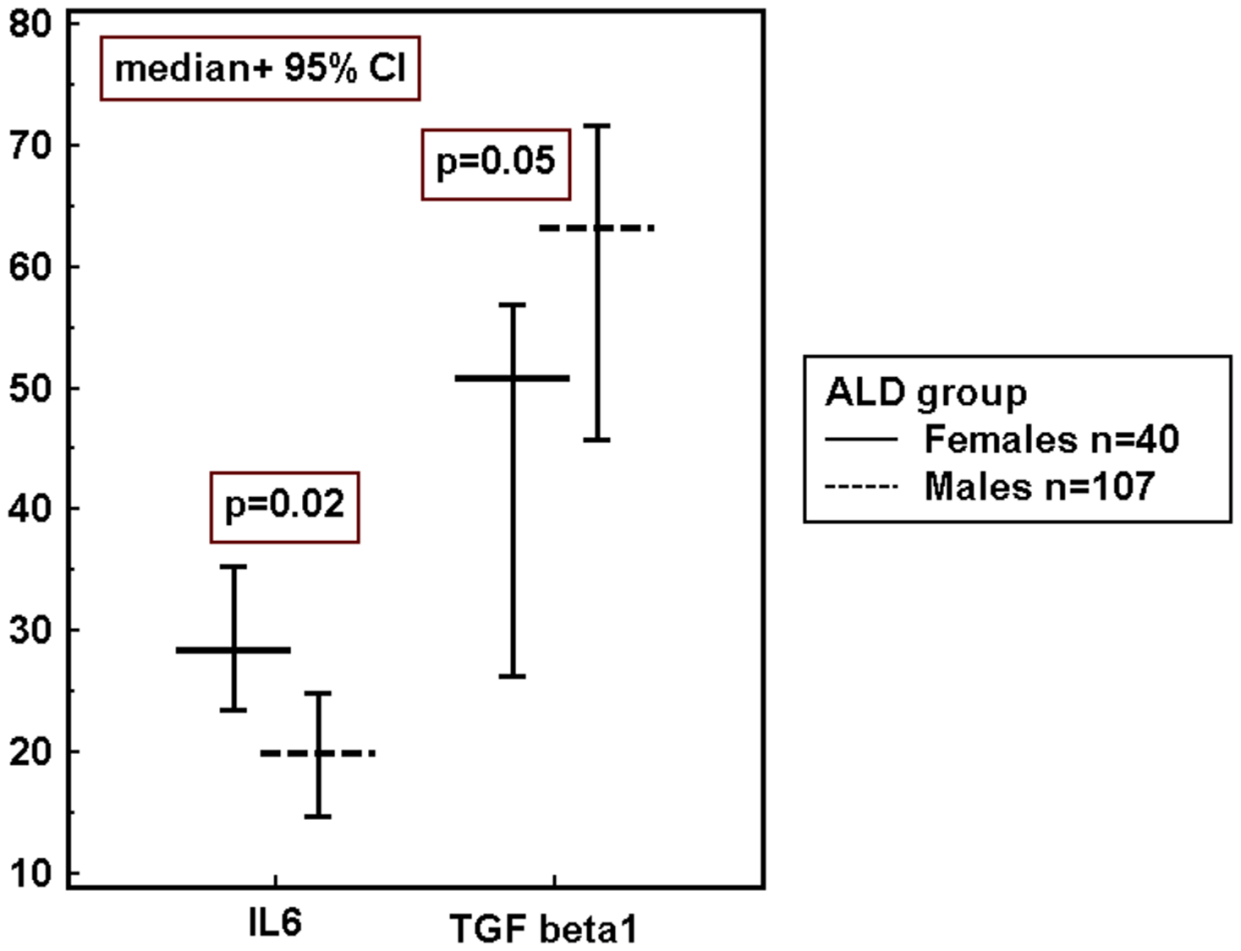
Gender- related differences in serum cytokine concentrations in ALD group. IL-6 pg/mL; TGF-beta1 ng/mL. ALD- alcoholic liver disease, 95% CI- Confidence Interval, IL- interleukin, TGF- Transforming Growth Factor, p- level of significance.

Subsequent analysis revealed no gender-related differences in the concentrations of inflammatory and immune biomarkers in the control group. However, as mentioned above, in patients with ALD significant gender-related differences in IL-6 and TGF-beta1 levels and the tendency to the higher percentage of Treg cells in females were observed (Table 1 and Fig 3).

### Inflammatory and immune regulator levels in ALD patients with different stages of liver dysfunction

**Fig 4:**
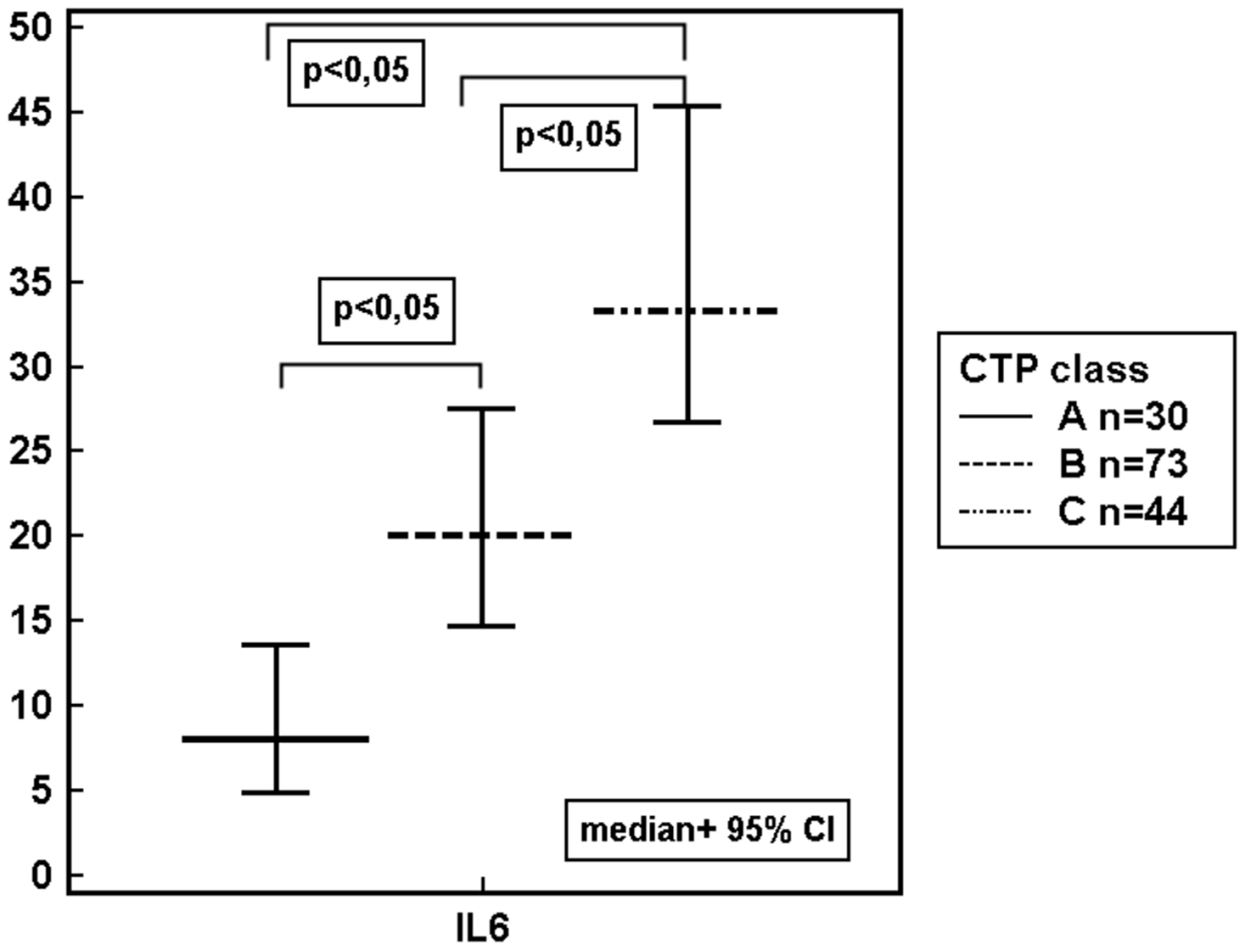
IL-6 concentrations (pg/mL) in ALD patients assigned according to the CTP class. ALD- alcoholic liver disease, 95% CI- Confidence Interval, CTP class- Child-Turcotte-Pugh class, IL- interleukin, p- level of significance.

Our study revealed, that the level of IL-6 were increasing in parallel to the grade of liver failure classified according to the CTP and MELD score (Table 3, Fig 4).

**Table 3:** Immune regulator levels in subgroups of ALD patients with different grade of liver failure.^a^

	**CTP class**						
	**class A (n = 30)**		**class B (n = 73)**		**class C (n = 44)**		
	median	95% CI	median	95% CI	median	95% CI	**p**
**IL-17A pg/mL**	10.43	8.79–12.74	11.12	9.39–14.66	10.30	8.31–14.77	0.90
**IL-1beta pg/mL**	0.65	0.27–2.36	0.73	0.43–1.30	1.13	0.14–4.18	0.54
**IL6 pg/mL**	8.03	4.94–13.65	20.07	14.73–27.51	33.23	26.72–45.39	**<0.0001**
**IL-23 pg/mL**	18.15	6.26–36.99	11.80	6.50–18.03	8.78	7.55–12.94	0.61
**TGF-beta1 pg/mL**	467.74	172.13–776.28	624.60	470.38–873.67	465.05	292.0–579.91	0.12
**Th17%**	0.94	0.71–1.14	1.06	0.83–1.21	0.68	0.65–0.96	0.12
**Treg %**	3.56	2.91–4.40	3.01	2.70–4.04	3.09	2.67–3.71	0.77
**Males**	25 (23.4%)		56 (52.3%)		26 (24.3%)		0.13
**Females**	5 (12.5%)		17 (42.5%)		18 (45%)		
	**MELD score**				
	**< 20 points (n = 117)**		**≥ 20 points (n = 30)**		
	median	95% CI	median	95% CI	**p**
**IL-17A pg/mL**	10.34	9.55–11.97	15.48	8.27–22.16	0.08
**IL-1beta pg/mL**	0.65	0.48–1.12	1.65	0.15–3.14	0.34
**IL-6 pg/mL**	19.88	14.51–24.69	29.37	26.36–42.20	**0.004**
**IL-23 pg/mL**	11.80	9.37–17.83	7.99	6.51–20.03	0.77
**TGF-beta1 pg/mL**	568.92	388.10–716.12	471.97	302.83–635.24	0.53
**Th17%**	0.90	0.71–1.04	0.98	0.69–1.64	0.14
**Treg %**	3.49	2.99–3.73	2.70	2.21–3.37	0.15
**Males**	85 (79.4%)		22 (20.6%)		0.88
**Females**	32 (80.0%)		8 (20.0%)		

^a^ ALD- alcoholic liver disease, CI- Confidence Interval, CTP class- Child-Turcotte-Pugh class, IL- interleukin, MELD- Model for End-Stage Liver Disease, p- level of significance, TGF- Transforming Growth Factor, Th- T helper cells; Treg- regulatory T cells.

Furthermore, the highest correlation was found between IL-6 and two parameters of liver function: positive for INR (S1 Fig) and inverse for serum albumin level (S2 Fig) (Rho 0.339; p<0.0001 and Rho—0.599; p<0.0001; respectively). A weak correlation between IL-1beta and AP, as well as IL-1beta and GGT was observed (Rho—0.20; p = 0.02; Rho—0.20; p = 0.01; respectively). Moreover, IL-6 correlated with all traditional indicators of inflammation: CRP level (Rho 0.48; p<0.0001) (S3 Fig), the white blood cell (Rho 0.19; p = 0.02) and neutrophil count (Rho 0.19; p = 0.02), and NLR (Rho 0.24; p = 0.004).

**Fig 5:**
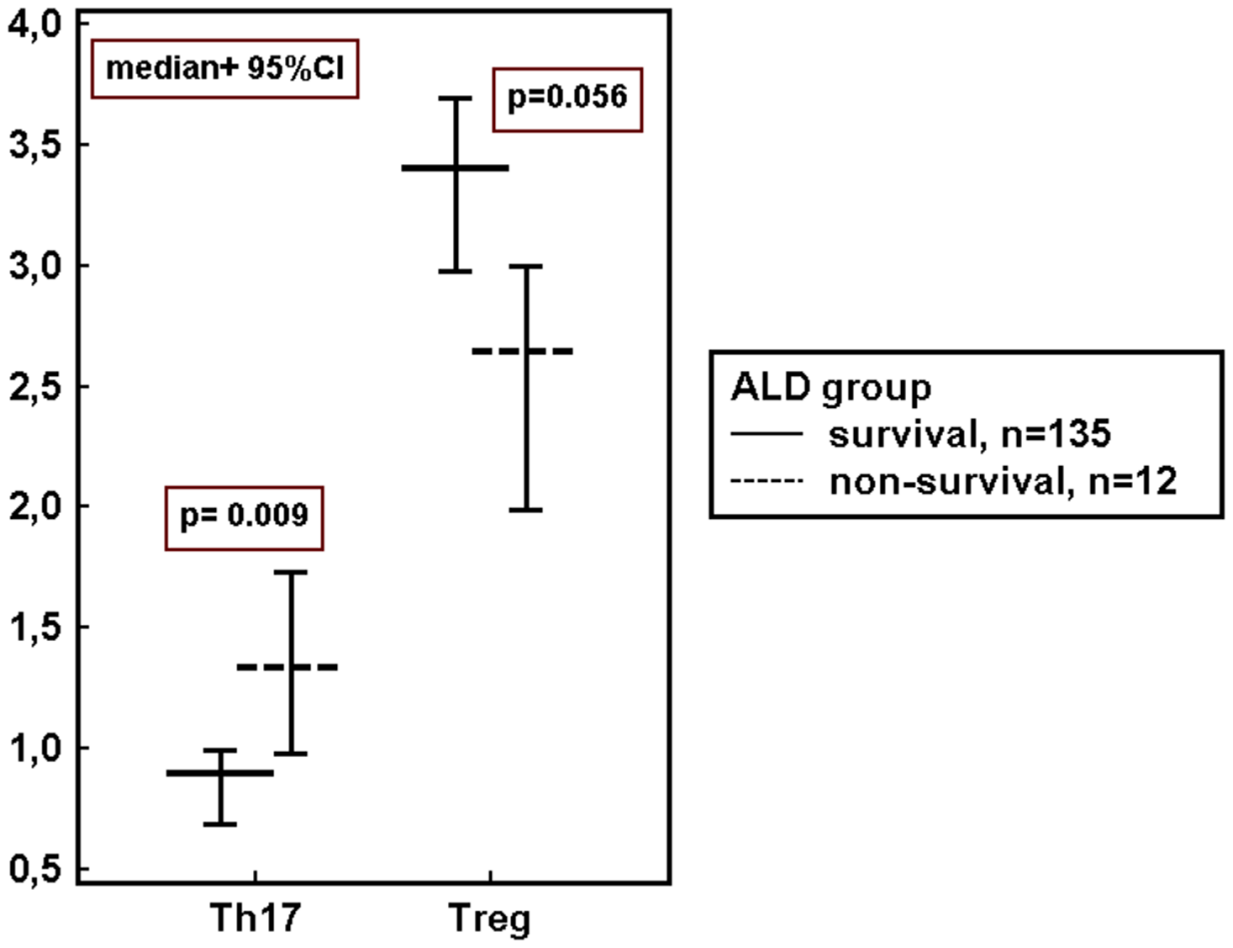
Peripheral blood frequencies (%) of Th17 and Treg cells in ALD survivors and non-survivors. ALD- alcoholic liver disease, 95% CI- Confidence Interval, p- level of significance, Th- T helper cells, Treg- regulatory T cells.

Then, we investigated concentrations of immune variables in ALD patients assigned according to the presence of the disease complications. Up-regulation of IL-6 was observed in subgroups with all studied ALD complications. Multiple pro-inflammatory regulators were increased in the blood of patients with ascites, hepatic encephalopathy and non-survivors in comparison to patients without these complications. Immune imbalance with significantly higher frequency of Th17 cells and the significantly lower percentage of Tregs in peripheral blood was found in non-survivors. The results are summarized in Table 4 and Fig 5.

**Table 4:** Immune regulator levels in ALD patients assigned according to the presence of the disease complications.^a^

	**Ascites**				
	**absent (n = 58)**		**persent (n = 89)**		
	median	95% CI	median	95% CI	**p**
**IL-17A pg/mL**	11.69	9.75–19.383	10.19	8.41–12.04	**0.053**
**IL-1beta pg/mL**	0.47	0.25–1.54	0.97	0.59–2.33	0.48
**IL-6 pg/mL**	10.47	7.54–14.35	29.39	25.12–35.92	**<0.0001**
**IL-23 pg/Ml**	18.15	11.98–28.87	7.99	6.45–10.80	**0.006**
**TGF-beta1 pg/mL**	705.15	469.09–854.83	499.34	334.10–617.17	0.25
**Th17%**	1.08	0.93–1.15	0.85	0.65–0.93	**0.04**
**Treg %**	3.20	2.84–3.63	3.31	2.83–3.74	0.44
**Males**	49 (45.8%)		58 (54.2%)		**0.02**
**Females**	9 (22.5%)		31 (77.5%)		
	**Hepatic encephalopathy**				
	**absent (n = 127)**		**present (n = 20)**		
	median	95% CI	median	95% CI	p
**IL-17A pg/mL**	10.34	9.52–12.02	20.11	8.99–24.15	**0.07**
**IL-1beta pg/mL**	1.10	0.59–1.65	0.07	0.00–0.93	**0.03**
**IL-6 pg/mL**	21.25	16.37–25.43	35.30	22.56–54.87	**0.03**
**IL-23 pg/mL**	10.80	7.99–14.54	12.55	6.30–31.72	0.53
**TGF-beta1 pg/mL**	554.32	458.13–706.09	471.97	263.57–707.05	0.28
**Th17%**	0.92	0.78–1.05	0.82	0.62–1.20	0.75
**Treg %**	3.37	2.91–3.67	3.03	2.48–3.59	0.74
**Males**	91 (85.0%)		16 (15.0%)		0.59
**Females**	36 (90.0%)		4 (10.0%)		
	**90-day disease outcome**				
	**survivors (n = 135)**		**non-survivors (n = 12)**		
	median	95% CI	median	95% CI	**p**
**IL-17A pg/mL**	11.12	9.85–12.93	9.09	6.93–11.30	0.27
**IL-1beta pg/mL**	0.73	0.50–1.50	0.58	0.09–5.37	0.82
**IL-6 pg/mL**	20.49	16.23–26.47	41.20	24.42–91.76	**0.006**
**IL-23 pg/mL**	11.80	9.37–16.14	5.56	2.45–21.46	0.23
**TGF-beta1 pg/mL**	554.32	388.10–705.15	499.34	321.83–887.53	0.86
**Th17%**	0.90	0.69–0.99	1.33	0.98–1.73	**0.009**
**Treg %**	3.40	2.98–3.69	2.64	1.99–2.99	**0.056**
**Males**	99 (92.5%)		8 (7.5%)		0.73
**Females**	36 (90.0%)		4 (10.0%)		

^a^ ALD- alcoholic liver disease, CI- Confidence Interval, IL- interleukin, p- level of significance, TGF- Transforming Growth Factor, Th- T helper cells; Treg- regulatory T cells.

In subgroups with oesophageal varices (n = 87) and renal impairment (n = 22), IL-6 was the only biomarker which concentrations significantly differed in comparison to ALD individuals without aforementioned complications (median; 95% CI: 24.63; 18.65–28.30 versus 15.92; 9.79–24.82; p = 0.04; and 31.14; 26.44–108.34 versus 20.07; 15.68–24.62; p = 0.003, respectively). No gender-related differences in the prevalence of both types of ALD complications were observed.

All traditional (Table 1) and immune variables (Table 2) were checked in univariate analysis for a possible association with the advanced liver dysfunction (MELD ≥20) and ALD complications in the studied cohort. For significant ones, the areas under the curve (AUCs) were assessed and their diagnostic accuracy was compared (Table 5).

**Table 5:** Comparison of the diagnostic accuracy (AUC) of single variables in the diagnosis of advanced liver dysfunction (MELD≥20) and ALD complications.^a^

Complication of ALD	Variable	p value	AUC(95% CI)	SE
MELD≥20	IL-6	0.0008	0.67(0.57–0.78)	0.05
	CRP	0.004	0.61(0.53–0.69)	0.06
	RBC	0.003	0.67(0.60–0.75)	0.05
	WBC	0.0003	0.66(0.58–0.73)	0.06
	Ascites	0.003	0.65(0.57–0.72)	0.05
	HE	<0.0001	0.67(0.59–0.74)	0.06
Ascites	IL-17A	0.049	0.60(0.50–0.69)	0.05
	IL-6	<0.0001	0.77(0.68–0.85)	0.04
	IL-23	0.004	0.63(0.54–0.73)	0.05
	Th17	0.041	0.60(0.50–0.69)	0.05
	Albumin	<0.0001	0.82(0.75–0.88)	0.04
	ALT	0.0001	0.71(0.63–0.78)	0.04
	AST	0.003	0.61(0.53–0.68)	0.05
	INR	<0.0001	0.81(0.74–0.87)	0.04
	RBC	0.002	0.66(0.58–0.74)	0.04
	WBC	0.008	0.60(0.52–0.67)	0.04
Hepatic encephalopathy	IL-1beta	0.014	0.65(0.53–0.77)	0.06
	IL-6	0.016	0.66(0.53–0.79)	0.07
	AP	0.006	0.65(0.57–0.73)	0.07
	Albumin	0.005	0.69(0.60–0.76)	0.05
	Bilirubin	0.0001	0.77(0.70–0.83)	0.05
	INR	0.0001	0.74(0.66–0.80)	0.06
	PLT	0.035	0.63(0.55–0.71)	0.06
	Ascites	0.012	0.65(0.57–0.72)	0.06
Renal impairment (crea>1.3mg/dL)	IL-6	0.0007	0.71(0.59–0.83)	0.07
	Albumin	0.034	0.65(0.57–0.73)	0.06
	AST	0.042	0.60(0.52–0.68)	0.06
	AP	0.030	0.68(0.59–0.75)	0.07
	Na	0.012	0.59(0.51–0.67)	0.08
	CRP	0.001	0.71(0.64–0.78)	0.06
	WBC	0.011	0.69(0.61–0.76)	0.05
	RBC	0.031	0.69(0.61–0.76)	0.06
Poor 90-day outcome (non-survival)	IL-6	0.009	0.79(0.71–0.85)	0.06
	Th17	0.0001	0.73(0.61–0.84)	0.06
	Treg	0.012	0.68(0.59–0.75)	0.07
	Bilirubin	0.0004	0.76(0.69–0.83)	0.06
	Albumin	0.0004	0.82(0.75–0.88)	0.06
	Na	0.003	0.75(0.68–0.82)	0.08
	AP	0.024	0.64(0.56–0.72)	0.10
	INR	0.009	0.73(0.66–0.80)	0.06
	Ascites	<0.0001	0.73(0.69–0.77)	0.02

^a^ ALD- alcoholic liver disease; ALT- alanine aminotransferase; AP- alkaline phosphatase; AST- aspartate aminotransferase; AUC- area under the ROC curve, CI- Confidence Interval, CRP- C-reactive protein; HE- hepatic encephalopathy, IL- interleukin; INR- International Normalized Ratio; Na- sodium; PLT- platelets; RBC- red blood cells; SE- Standard Error, Th- T helper cells; Treg- regulatory T cells; WBC- white blood cells.

Applications of ROC curves include assessment of the effectiveness of continuous diagnostic markers in distinguishing between diseased and non-diseased individuals. As reported elsewhere, AUC has a real clinical significance when its value exceeds 0.7 and AUC values between 0.8 and 0.9 demonstrate excellent diagnostic accuracy [30, 31].

In our study, the highest AUCs were found for IL-6 in ascites (0.77) and non-survivor (0.79) subgroups, demonstrating moderate discriminatory power and, therefore potential utility as a diagnostic test in evaluation of ALD complications. However, AUC values for the traditional liver function parameter, this is albumin concentrations, performed better in both aforementioned subgroups (0.82 and 0.82, respectively).

### Selection of independent predictors of advanced liver dysfunction and/or ALD complications

Finally, multivariable logistic regression was applied in order to select independent predictors of advanced liver failure and ALD complications. Multivariable analysis of significant, immune factors revealed independent association of IL-6 with the ascites development, as well as the poor 90 day prognosis. Furthermore, we also found the borderline significance of Th17 impact on the survival (Table 6).

**Table 6:** Immune predictors of advanced liver dysfunction (MELD≥20) and ALD complications.^a^

Complication of ALD	Variable	Univariable analysis	Multivariable analysis
		p	p
MELD≥20	IL-6	0.0008	
Ascites	IL-17A	0.049	NS
	**IL-6**	<0.0001	**0.0009**
	IL-23	0.004	NS
	Th17	0.041	NS
Hepatic encephalopathy	IL-1beta	0.014	NS
	IL-6	0.016	NS
Renal impairment (crea>1.3mg/dL)	IL-6	0.0007	
Poor 90-day outcome(non-survival)	**IL-6**	0.009	**0.029**
	**Th17**	0.0001	**0.054**
	Treg	0.012	NS

^a^ ALD- alcoholic liver disease, IL- interleukin, MELD- Model for End-Stage Liver Disease, NS- not significant, p- level of significance, Th- T helper cells; Treg- regulatory T cells.

When immune factors were adjusted to significant, traditional indicators of liver failure and analyzed together, only two variables revealed their independent association with ALD complications. Plasma IL-6 and sodium levels influenced the mortality rate over a 90 day follow-up. The predictive power (AUC) of the complex statistical model constructed from the two significant parameters occurred to be superior to the power of either parameter alone (Table 7).

**Table 7:** Independent predictors of poor ALD outcome (multivariable analysis).^**a**^

Complication of ALD	Variable	p value	Adjusted OR (95% CI)	AUC (95% CI)	SE
Poor 90-day outcome (non-survival)	IL-6	0.009	1.01 (1.00–1.01)	**0.92** (0.86–0.96)	0.02
	Na	0.001	0.80 (0.70–0.91)		

^a^ ALD- alcoholic liver disease, AUC- area under the ROC curve, CI- Confidence Interval, IL- interleukin, Na- sodium, OR- Odds ratio, p- level of significance, SE- standard error.

High concentrations of IL6 turned out to be the only independent predictor of non-survival among studied immune regulators. No independent association of any other biomarker with any complication of ALD could be confirmed.

## Discussion

Results of the present study support concepts that excessive alcohol consumption induces systemic inflammatory activation with a pro-inflammatory cytokine storm which may affect multiple organs and tissues. Pro-inflammatory tumor necrosis factor alpha (TNF-alpha) has already been demonstrated to be one of the key factors in the pathogenesis of ALD [32]. Our study revealed significant over-expression of pro-inflammatory, Th17 lymphocyte-related cytokines: IL-17A, IL-1beta and IL-6 in coexistence with significant down-regulation of Treg lymphocyte-related TGF-beta1 in peripheral blood of ALD group in comparison to healthy controls. These data are consistent with results obtained by Lemmers et al. [33] who first reported the involvement of the IL-17 pathway in human alcoholic liver disease. Elevated IL-17 concentrations have been also reported in other liver diseases: autoimmune hepatitis, HBV and HCV infections, and primary biliary cirrhosis [34–36]. Surprisingly, no difference in plasma IL-23 levels, which is crucial for the differentiation and maintenance of Th17 cells, was found. Nevertheless, it was in agreement with results obtained by Stoy et al. [37] who also did not observed any changes in the blood concentrations of IL-23 in patients with alcoholic hepatitis. On the other hand the similar cytokine profile, this is decreased levels of TGF-beta1 in coexistence with increased IL-17 concentrations, was described by Xing et al. [38] in patients with lupus nephritis. Rollnik et al. [39] reported reduced TGF-beta1 levels in serum in multiple sclerosis. Decreased concentrations of TGF-beta1 in inflammatory disorders may be explained by its other characteristics. In addition to the promotion of Treg cells differentiation, it can inhibit the secretion of many cytokines (eg. interferon-gamma, TNF-alpha), as well as the expression of cytokine receptors, (eg. IL-2 receptor), and as a consequence down-regulate the activity of immune cells [40]. On the other hand, decreased TGF-beta1 concentrations (also found in our patients with ALD) may contribute to perpetuation of inflammatory reactions.

Our data suggest that IL-6 have the highest diagnostic and prognostic value among all studied biomarkers. Its increased concentrations were associated with all major ALD complications (ascites, hepatic encephalopathy, oesophageal varices, renal impairment and mortality). We also observed isolated over-expression of IL-6 increasing in parallel to the severity of liver dysfunction defined according to the criteria of the CTP and MELD (Table 3, Fig 4). No other studied inflammatory and/or immune biomarker revealed association with above scores. Furthermore, IL-6, as well as sodium levels, turned out to be an independent predictor of poor 90-day prognosis (non-survival). The prognostic value of sodium in cirrhotics is well recognized [41, 42]. Our results remain in accordance with Sheron et al. report [43] that found a correlation between elevated plasma IL-6 and increased severity and mortality in alcoholic hepatitis. Nevertheless, the role of IL-6 in ALD is complex and not quite clear. On the one hand, IL-6 may promote human Th17 differentiation and IL-17 production contributing to ethanol-induced liver inflammation. On the other hand, several recent experimental studies in animal models have suggested that IL-6 plays a crucial role in the protection against alcohol-induced liver damage [44, 45]. It exerts this action via the activation of signal transducer and activator of transcription 3 (STAT3) and the subsequent induction of several hepatoprotective genes in hepatocytes [46, 47]. Consistent with this, elevated IL-6 concentrations in our cohort might in part reflect mechanisms ameliorating alcoholic liver injury. All together, targeting IL-6 signaling represent a tempting option and might be a potential therapeutic strategy in ALD. Nevertheless, we have to keep in mind the anti-TNF-alpha experience where the initial results with the anti-TNF agent infliximab were also particularly promising [48], but subsequent increase in mortality and a high rate of infection led to trials termination [49].

The altered profile of studied cytokines suggested that defects in inflammatory regulation in the course of ALD might derive from impaired balance of T helper subsets. Our subsequent assessment of their blood frequencies revealed the systemic Th17/Treg imbalance present in the most severely ill patients who died within 90-day follow up. Recently, Ma et al. [50] have reported that oxidative stress in fatty liver induced apoptosis of Treg, reduced their hepatic content, and led to decreased suppression of inflammatory responses. It is likely that Th17/Treg imbalance might contribute to the protraction of inflammation leading to the fatal disease outcome in our cohort. Nevertheless, multivariable logistic regression failed to show both Th17 and Treg cells independent impact on mortality in our cohort. The present study suggests that the imbalance of Th17/Treg cells, although present in the most severe stage of ALD, may not play a decisive role early in the disease course. Alterations in the Th17/Treg balance have also been confirmed in experimental liver fibrosis in mice [51], as well as in several autoimmune and inflammatory diseases and cancers [52–54].

Interestingly, analysis of data by gender showed the presence of significant differences in concentrations of some inflammatory and immune biomarkers between men and women with ALD. They were not found in healthy subjects. We observed significantly higher neutrophil counts and IL-6 concentrations, but lower TGF-beta1 levels and a tendency to the higher frequency of Treg cells in females in comparison with males. We are tempted to speculate that gender-related differences in the inflammatory and immune response in the course of ALD might influence different susceptibility to liver injury in both sexes.

Men and women in our ALD cohort differed significantly with respect to their liver function parameters (ALT and GGT activities, INR). Since females presented with more severe liver dysfunction as indicated by higher CTP and MELD scores (what was in line with their higher GGT and INR), their lower ALT activities in this context might be explained by a reduced grade of inflammation in the more advanced disease stage. Our results are consistent with epidemiological data indicating that women develop alcohol-related liver cirrhosis at earlier age [55, 56]. Therefore, it is likely that in our study where both gender subgroups did not differ significantly with respect to their age (p> 0.05), the extent of hepatic damage in females was greater.

Anemia and ascites were also more prevalent in women with ALD than in men. As a rule, women of reproductive age are at increased risk of iron deficiency anemia. In addition, it might be explained in part by the chronic inflammatory response in ALD. Moreover, a protein nutrition status might have an impact on ascites formation in both sexes, although we could not confirm significant differences in peripheral blood albumin levels.

We did not find significant correlations between the number of drinks per day and cytokine concentrations and/or inflammatory parameter levels. It is likely that there is not a simple effect of an amount of alcohol consumption on the immune response. Although it is the most potent factor, ALD mechanisms seem to be more complicated and several host and environmental factors may account for variations in the intensity of liver inflammation as well as susceptibility to alcohol-induced liver injury [57–60]. Our results are consistent with previous reports indicating that some heavy drinkers do not suffer from liver injury [61, 62]. These aspects of ALD pathogenesis require further investigations.

There are some limitations of the present study. The results obtained in the single-center trial need to be confirmed by future multicenter studies. Such validation may help to eliminate possible errors resulting from research techniques and subjective differences in the selection of the study population. Alcohol consumption was self-reported in the study as drinks per day, week, and/or month (one drink contained 10 g of ethanol). Since the volume and alcohol concentration of beverages are not easy to estimate, the real ethanol intake may lack precision [63]. Also Stockwell and Stirling [64] reported that most individuals are not able to accurately assess the volume and power of one drink. In countries like Poland, alcohol intake patterns vary considerably by regions and beverage type, so special attention should be paid to the assessment of drink types and sizes for accurate alcohol consumption estimation.

## Conclusions

Results obtained in the present study correspond to the general belief in the presence of peripheral inflammatory activation during the course of ALD. We provide evidence that IL-6 revealed the highest diagnostic and prognostic value among all studied immune biomarkers. Its systemic concentrations were related to the severity of liver dysfunction, development of ALD complications and 90-day survival. The presence of systemic Th17/Treg imbalance might be a poor prognostic indicator associated with the fatal ALD course. Moreover, it seems that gender-related differences in the inflammatory and immune response might influence the susceptibility to alcohol-associated liver injury in males and females. Our results require further confirmation in multicenter studies.

## Supporting Information

S1 Fig Correlation between INR and serum IL-6 concentrations (pg/mL) in 147 patients with ALD. Rank correlation test. A logarithmic transformation was used for both variables. ALD- alcoholic liver disease, 95% CI- Confidence Interval, IL- interleukin, p- level of significance, Rho- Spearman's correlation coefficient.
(TIF)

S2 Fig Correlation between serum albumin (g/dL) and IL-6 (pg/mL) concentrations in 147 patients with ALD. Rank correlation test. A logarithmic transformation was used for both variables. ALD- alcoholic liver disease, 95% CI- Confidence Interval, IL- interleukin, p- level of significance, Rho- Spearman's correlation coefficient.
(TIF)

S3 Fig Correlation between serum CRP levels (mg/L) and IL-6 concentrations (pg/mL) in 147 patients with ALD. Rank correlation test. A logarithmic transformation was used for both variables. ALD- alcoholic liver disease, 95% CI- Confidence Interval, CRP- C-reactive protein, IL- interleukin, p- level of significance, Rho- Spearman's correlation coefficient.
(TIF)
