# Supplementary figures and images for: Prognostic Significance of the Systemic Inflammatory and Immune Balance in Alcoholic Liver Disease with a Focus on Gender-Related Differences
Source: PLoS One. 2015 Jun 24;10(6):e0128347. doi: 10.1371/journal.pone.0128347 (PMC4480424; doi:10.1371/journal.pone.0128347)

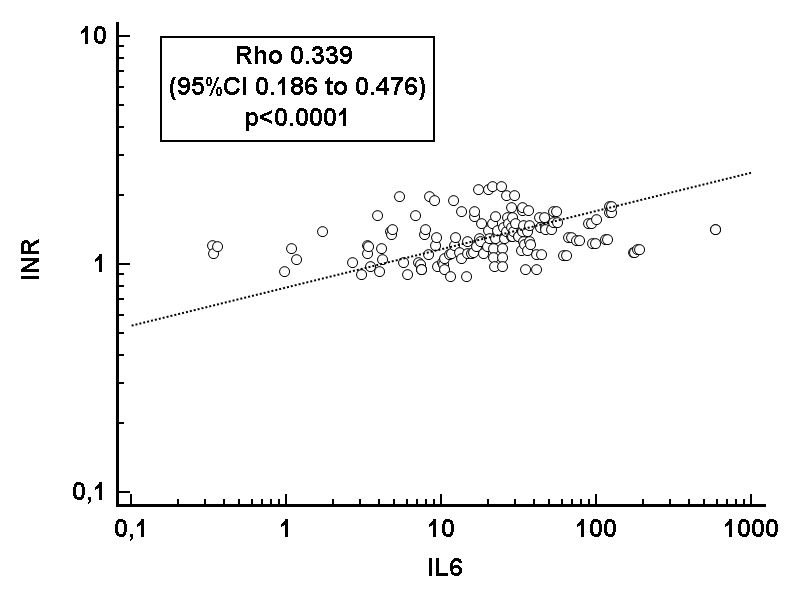

Supplement: S1 Fig — Rank correlation test. A logarithmic transformation was used for both variables. ALD- alcoholic liver disease, 95% CI- Confidence Interval, IL- interleukin, p- level of significance, Rho- Spearman's correlation coefficient. (TIF) [file pone.0128347.s001.tif]

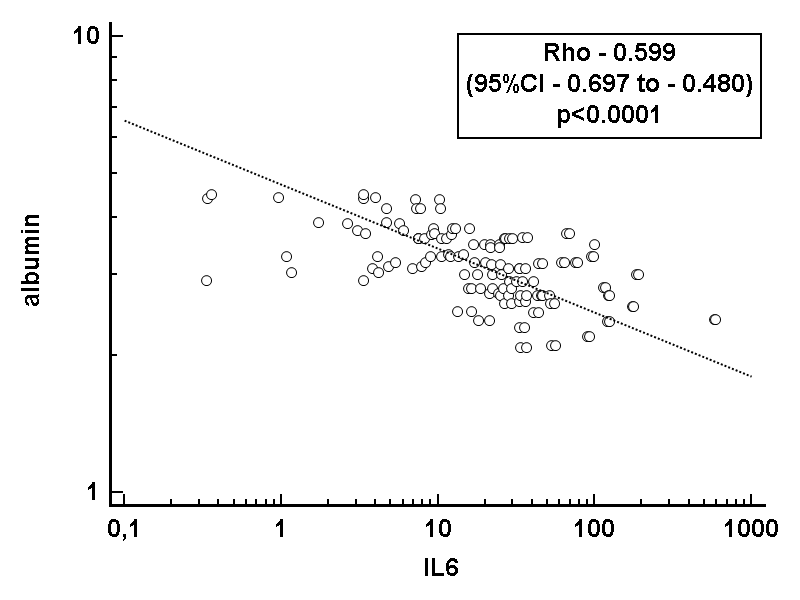

Supplement: S2 Fig — Rank correlation test. A logarithmic transformation was used for both variables. ALD- alcoholic liver disease, 95% CI- Confidence Interval, IL- interleukin, p- level of significance, Rho- Spearman's correlation coefficient. (TIF) [file pone.0128347.s002.tif]

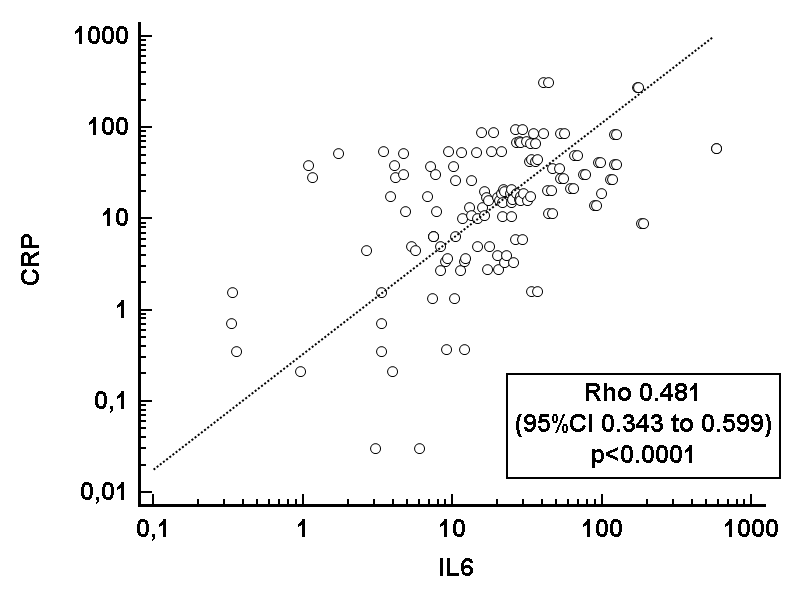

Supplement: S3 Fig — Rank correlation test. A logarithmic transformation was used for both variables. ALD- alcoholic liver disease, 95% CI- Confidence Interval, CRP- C-reactive protein, IL- interleukin, p- level of significance, Rho- Spearman's correlation coefficient. (TIF) [file pone.0128347.s003.tif]
